# The function of BTG3 in colorectal cancer cells and its possible signaling pathway

**DOI:** 10.1007/s00432-017-2561-9

**Published:** 2017-12-21

**Authors:** Chi Lv, Heling Wang, Yuxin Tong, Hongzhuan Yin, Dalu Wang, Zhaopeng Yan, Yichao Liang, Di Wu, Qi Su

**Affiliations:** 1 Department of General Surgery, Shengjing Hospital Affiliated to China Medical University, Shenyang City, Liaoning Province 110004 People’s Republic of China; 2 Department of General Surgery, General Hospital of Shenyang Military Region, Shenyang, People’s Republic of China; 3 Medical Research Center, Shengjing Hospital of China Medical University, Shenyang, People’s Republic of China

**Keywords:** BTG3, Colorectal cancer, Prognosis, Biological behavior, Genomic microarray

## Abstract

**Purpose:**

B-cell translocation gene 3 (BTG3) has been identified as a candidate driver gene for various cancers, but its specific role in colorectal cancer (CRC) is poorly understood. We aimed to investigate the relationship between expression of BTG3 and clinicopathological features and prognosis, as well as to explore the effects and the role of a possible BTG3 molecular mechanism on aggressive colorectal cancer behavior.

**Methods:**

BTG3 expression was assessed by immunohistochemistry (IHC) on specimens from 140 patients with CRC. The association of BTG3 expression with clinicopathological features was examined. To confirm the biological role of BTG3 in CRC, two CRC cell lines expressing BTG3 were used and BTG3 expression was knocked down by shRNA. CCK-8, cell cycle, apoptosis, migration, and invasion assays were performed. The influence of BTG3 knockdown was further investigated by genomic microarray to uncover the potential molecular mechanisms underlying BTG3-mediated CRC development and progression.

**Results:**

BTG3 was downregulated in colorectal cancer tissues and positively correlated with pathological classification (*p* = 0.037), depth of invasion (*p* = 0.016), distant metastasis (*p* = 0.024), TNM stage (*p* = 0.007), and overall survival (OS) and disease-free survival (DFS). BTG3 knockdown promoted cell proliferation, migration, invasion, relieved G2 arrest, and inhibited apoptosis in HCT116 and LoVo cells. A genomic microarray analysis showed that numerous tumor-associated signaling pathways and oncogenes were altered by BTG3 knockdown. At the mRNA level, nine genes referred to the extracellular-regulated kinase/mitogen-activated protein kinase pathway were differentially expressed. Western blotting revealed that BTG3 knockdown upregulated PAK2, RPS6KA5, YWHAB, and signal transducer and activator of transcription (STAT)3 protein levels, but downregulated RAP1A, DUSP6, and STAT1 protein expression, which was consistent with the genomic microarray data.

**Conclusions:**

BTG3 expression might contribute to CRC carcinogenesis. BTG3 knockdown might strengthen the aggressive colorectal cancer behavior.

## Introduction

Colorectal cancer (CRC) is the second leading cause of cancer mortality worldwide and the third most frequently diagnosed malignancy (Cassidy and Syed 2017). The CRC incidence and mortality rates have declined in recent decades, largely attributed to the contribution of preventive screening and early detection (Siegel et al. 2014). However, rapid increases in prevalence have been noted in previously low-risk countries, such as China, and have been ascribed to particular increases in the prevalence of obesity and physical inactivity (Varghese and Shin 2014). CRC places a financial and economic burden worldwide, especially in developing countries (Alefan et al. 2017). Thus, effective therapies for CRC are needed urgently. To achieve this goal, it is important to explore the genetic and molecular abnormalities underlying CRC.

B-cell translocation gene 3 (BTG3) belongs to the B-cell translocation gene (BTG)/transducer of the ErbB2 family, which comprises structurally related proteins that appear to have antiproliferative properties (Winkler 2010). BTG3 is reportedly a candidate tumor suppressor, as it inhibits cell proliferation and migration, and regulates cell cycle progression in several tumors, such as gastric cancer (Ren et al. 2015) and esophageal adenocarcinoma (Gou et al. 2015; Du et al. 2015). Previous studies have demonstrated that BTG3 expression is downregulated in many cancers, including prostate cancer, renal carcinoma, and hepatocellular carcinoma (Majid et al. 2010, 2009; Lv et al. 2013). Moreover, BTG3 expression may predict survival and prognosis in patients with gastric cancer and ovarian carcinoma (Ren et al. 2015; Deng et al. 2013). However, BTG3 status in patients with CRC and its causal role in the development of CRC remain elusive.

To clarify the biological functions of BTG3 in CRC, we first explored the expression of BTG3 in CRC clinical samples and investigated its clinicopathological features and prognostic value. Then, we evaluated the effects of depleting BTG3 on cell proliferation, the cell cycle distribution, apoptosis, migration, and invasion of CRC cells. Finally, a microarray analysis revealed that multiple pathways critical for CRC development and progression were regulated and the expression of several signaling related genes was altered by BTG3 knockdown, providing valuable insight into the molecular mechanisms underlying BTG3-mediated biological functions in CRC.

## Materials and methods

### Colon cancer clinical tissue specimens and cell culture

A total of 140 cases of paraffin specimens of primary colon cancer were obtained by surgical resection from the Department of Pathology, Shengjing Hospital affiliated with China Medical University from 2013 to 2014. Patients were not pretreated with radiotherapy or chemotherapy prior to surgery. The patients were followed up, and their complete clinical data were collected. Overall survival (OS) was defined as the interval between the dates of surgery and death. Disease-free survival (DFS) was defined as the interval between the dates of surgery and disease recurrence; if disease recurrence was not diagnosed, patients were censored at the date of death or last follow-up.

Histological types were assigned according to the criteria of the National Comprehensive Cancer Network classification system. Matched adjacent normal colorectal tissues from the macroscopic tumor margin (at least more than 5 cm away from the tumor and histologically confirmed) were isolated at the same time and used as the control. This study was approved by the Ethics Committee of the Affiliated Shengjing Hospital of China Medical University. The ethics number was 2016PS274K. Informed consent was obtained from all patients prior to tissue acquisition.

The HT29, HCT116, SW480, SW620, LoVo, and RKO CRC cell lines were obtained from Cell Bank of Chinese Academy of Sciences (Shanghai, China). They were cultured in RPMI 1640, DMEM, or Ham’s F12K medium (Hyclone, Logan, UT, USA) supplemented with 10% fetal bovine serum (FBS), 100 units/mL penicillin, and 100 μg/mL streptomycin (Hyclone) and maintained in an incubator with a humidified atmosphere of 95% air and 5% CO_2_ at 37 °C.

### Immunohistochemistry

An immunohistochemical analysis was performed on 4-mm, formalin-fixed, paraffin-embedded tissue sections according to the following procedures. Briefly, consecutive sections were deparaffinized in xylene, rehydrated in a graded ethanol series, and submerged in EDTA antigenic retrieval buffer for 15 min in a microwave oven. The sections were treated with 3% hydrogen peroxide in absolute methanol for 20 min to block endogenous peroxidase activity. 5% bovine serum albumin was applied for 15 min to prevent non-specific binding. The sections were incubated with a rabbit polyclonal antibody against human BTG3 (1:100; Abcam, Cambridge, MA, USA) overnight at 4 ℃. After the incubation with secondary antibody, the visualization signal was developed with 3,3′-diaminobenzidine tetrachloride. The primary antibody was omitted for the negative control.

BTG3 immunostaining densities were quantitatively assessed with NIS-Elements BR 3.0 (Nikon, Tokyo, Japan). In brief, the sections were placed on a microscope (Nikon E800), and the images were transferred from a digital camera (Nikon 80i) to a computer. Three visual fields were randomly inspected on all slides under high-power magnification. The mean optical density (MOD) of the positive areas was measured. The results are expressed as the exact value of the relative optical density units.

### Recombinant lentiviral vector construction and cell infection

shRNA specifically targeting the BTG3 gene (NM_001130914.1) was designed and the lentivirus was constructed and prepared to deplete BTG3. Briefly, shRNA1 (target sequence: AGGAATGTATCGAGGGAAT) and shRNA2 (target sequence: TGAGAAATTGACCCTAATA) targeting human BTG3 were designed and the negative control sequence was as follows: TTCTCCGAACGTGTCACGT. Related stem-loop DNA oligonucleotides were synthesized, annealed, and inserted into lentiviral vector pGCSIL-GFP (GeneChem, Shanghai, China). The Lentivector Expression System (GeneChem) was used to produce lentivirus expressing BTG3 shRNA or the silencing negative control sequence shRNA.

### Infection of CRC cells with lentivirus

HCT116 and LoVo cells were plated in 12-well plates and infected with a lentivirus expressing BTG3 (shRNA1 and shRNA2) or the silencing negative control sequence shRNA was added according to the multiplicity of infection. After 72 h of infection, the cells were observed under a fluorescence microscope (MicroPublisher 3.3RTV; Olympus). After 120 h of infection, the cells were harvested to determine knockdown efficiency by western blot analysis.

### Western blot analysis

The cells were collected and lysed using ice-cold lysis buffer containing 1 mM protein inhibitor and 1 mM PMSF for 30 min on ice. The cell lysates were centrifuged at 12,000×*g* at 4 °C for 30 min, and the supernatants were collected. After the protein was quantified by Coomassie Brilliant Blue staining, 40 mg of protein was loaded in a loading buffer, resolved by 10% SDS-polyacrylamide gel electrophoresis, electrotransferred to PVDF membranes, and incubated overnight with primary antibody (STAT3, FOS, RPS6KA5, RAP1A, DUSP6, ATF4 from Abcam; STAT1, PAK2, YWHAB from CST; GAPDH from Santa Cruz Biotechnology, Santa Cruz, CA, USA; β-actin from ZSBIO, Beijing, China). Secondary antibody was applied, and the relative content of the target proteins was detected with an enhanced chemiluminescence reagent (SuperSignal West Pico; ThermoScientific, Rockford, IL, USA). β-actin was used as the loading control.

### Cell proliferation assay

About 2 × 10^3^ cells/wells were seeded on a 96-well plate. The Cell Counting Kit-8 (CCK-8) (Dojindo, Kumamoto, Japan) was employed to determine the number of viable cells over 5 days. In brief, 10 μL of CCK-8 solution was added to each well of the plate at different time points, and the plates were incubated for 4 h in an incubator and absorbance was measured at 450 nm. Each cell group was plated in three duplicate wells.

### Cell cycle analysis

About 1 × 10^6^ cells were trypsinized, washed twice with PBS, and fixed in cold 10 mL of ethanol overnight. Then, the cells were centrifuged to remove the ethanol, washed twice in PBS, and incubated with 100 μL RNase at 37 °C for 30 min. The cells were pelleted and resuspended in 400 μL propidium iodide and incubated at 4 °C in the dark for 30 min. Finally, flow cytometry was employed to examine the cell cycle distribution.

### In vitro apoptosis assay

About 2 × 10^5^ cells were harvested and stained with 7-AAD and FITC-labeled Annexin V (KGA1017; KeyGEN Biotech, Nanjing, China,) to detect phosphatidylserine externalization as an endpoint indicator of apoptosis according to the manufacturer’s instructions by flow cytometry using a BD FACSCalibur system (Becton Dickinson, Brea, CA, USA).

### Transwell chamber assays

About 8 × 10^4^ cells were resuspended in serum-free RPMI 1640 and seeded in the upper chamber (3422; Corning COSTAR, Corning, NY, USA) with Corning Matrigel (cat # 356243). The lower compartment of the chamber was filled with RPMI-1640 and 10% FBS as a chemoattractant. After a 37 °C incubation in 5% CO_2_ for 24 h, the cells on the membrane were scrubbed, washed with PBS, fixed in 100% methanol, and stained with Crystal Violet Staining Solution (Solarbio, Beijing, China) for 30 min. The procedures for the migration assay were the same as described above, except no Matrigel was used. The cells in the lower chamber were counted under a light microscope in five random visual fields (×200).

### Microarray processing and analysis

Total RNA from HCT116 cells infected with lentivirus expressing either NC/shRNA (*n* = 3) or BTG3/shRNA1 (*n* = 3) was extracted using Trizol reagent. Then, RNA quantity and quality were assessed with the Thermo NanoDrop 2000 and Agilent 2100 Bioanalyzer. Affymetrix human GeneChip primeview was used for microarray processing to determine the gene expression profile according to the manufacturer’s instructions (Affymetrix Inc., San Diego, CA, USA). In brief, reverse transcription, double-stranded DNA template transformation, in vitro transcription for mRNA synthesis, and biotin labeling were all conducted using the GeneChip 3′ IVT Expression Kit. Microarray hybridization, washing, and staining were performed using the GeneChip Hybridization Wash and Stain Kit. The arrays were scanned using the GeneChip Scanner 3000 to produce raw data. Significant differentially expressed genes between HCT116 cells treated with BTG3/shRNAs and HCT116 cells treated with NC/shRNAs were selected based on the following criteria: *p* value < 0.05 and absolute fold change > 1.3.

### IPA

All of the significantly differentially expressed genes were used to query the Ingenuity Pathway Analysis (IPA) system (Ingenuity Systems, Mountain View, CA, USA) and to compose a set of interactive networks considering canonical pathways, relevant biological interactions, cellular and disease processes, and molecular regulatory networks.

### Statistical analysis

The SPSS 23.0 statistical package was used (SPSS Inc. Chicago, IL, USA). Means were compared using Student’s *t* test or analysis of variance. Univariate analyses were performed using the Kaplan–Meier method and comparisons between survival curves were made with log-rank statistics. Cox multivariate analysis was used to determine the independent prognostic factors. The “minimum *p* value” approach (Wolfgang 2013; Budczies et al. 2012) was used to obtain the optimal cutoff value for the best separation between groups of patients in relation to OS or DFS. A two-tailed P value < 0.05 was considered significant.

## Results

### BTG3 is downregulated in human CRC tissues and correlated with clinicopathological parameters and prognosis

**Fig. 1:**
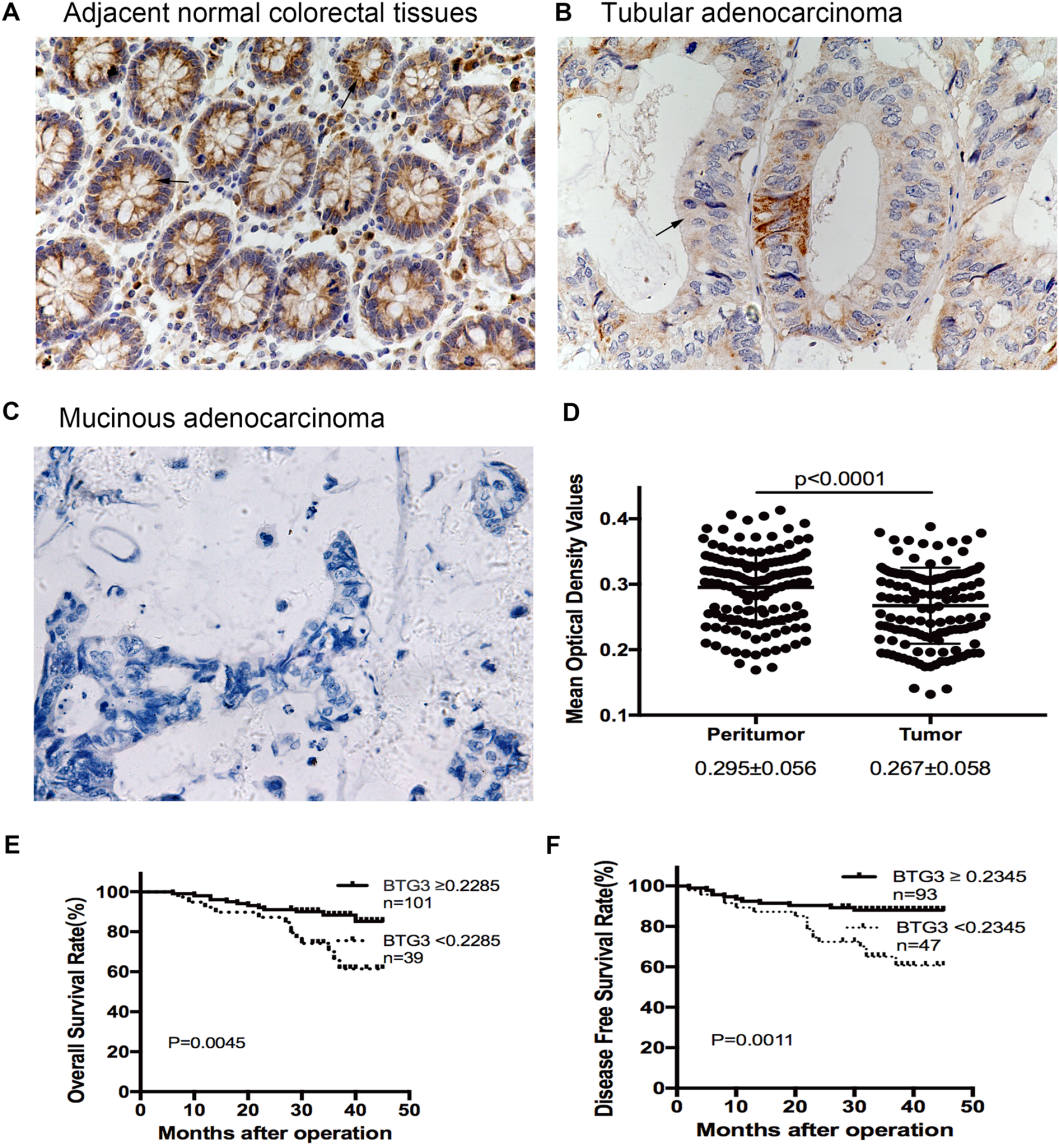
Expression of BTG3 in clinical paraffin-embedded CRC tissues and survival analysis of BTG3 expression in patients with CRC. **a** Strong positive expression of BTG3 in adjacent normal colorectal tissues (×400). **b** Moderate cytoplasmic BTG3 staining in adenocarcinoma tissues (×400). **c** Weak expression of BTG3 in mucinous adenocarcinoma tissues (×400). Arrows indicate the cytoplasmic BTG3 staining. **d** IHC analysis results of BTG3 expression in 140 patients with CRC. Mean optical density (MOD) values were significantly lower in CRC tissues than paired adjacent normal colorectal tissues (*p* < 0.0001). **e** Kaplan–Meier survival analysis for overall survival rate of patients with high and low BTG3 expression (*p* = 0.0045). **f** Kaplan–Meier survival analysis for disease-free survival rate of patients with high and low BTG3 expression (*p* = 0.0011)

Immunohistochemical staining (IHC) was performed in 140 paired paraffin-embedded samples to detect the expression of BTG3 and its clinicopathological characteristics in patients with CRC. Compared to the relatively stronger cytoplasmic expressions of BTG3 in adjacent normal colorectal tissues (Fig. 1a), BTG3 expressed in tumor tissues with mostly moderate to weak cytoplasmic staining (Fig. 1b, c). More important, the majority of adenocarcinoma tissues showed significantly higher cytoplasmic expression of BTG3 than mucinous adenocarcinoma (Fig. 1c). Consistent with these findings, the mean optical density of BTG3 expression in CRC tissues was significantly lower than that in adjacent normal tissue (*p* < 0.0001, Fig. 1d). Clinicopathological analyses showed that BTG3 expression was correlated with pathological classification (*p* = 0.037), depth of invasion (*p* = 0.016), distant metastasis (*p* = 0.024), and TNM stage (*p* = 0.007, Table 1). A survival analysis was conducted to assess the prognostic value of BTG3 expression. Using the “minimum P value” approach, the mean optical density (MOD) values of 0.2285 and 0.2345 were the best cutoff values for overall survival (OS) and disease-free survival (DFS). Follow-up information was available on 140 patients with CRC for 2–45 months (median = 34 months). A Kaplan–Meier analysis revealed that patients with CRC and relatively high BTG3 expression had significantly longer OS and DFS rates than those with low BTG3 expression (OS rate, *p* = 0.0045, Fig. 1e; DFS rate, *p* = 0.0011, Fig. 1f). The significant prognostic factors for both OS and DFS rates in the univariate analysis were BTG3 expression (*p* = 0.007 and *p* = 0.002), lymphatic invasion (*p* = 0.019 and *p* = 0.024), and TNM stage (*p* = 0.022 and *p* = 0.030, Table 2). Further analysis with a multivariate Cox proportional hazards model demonstrated that BTG3 expression (*p* = 0.005 and *p* = 0.016), together with lymphatic invasion (*p* = 0.041 and *p* = 0.047, Table 2) were strongly associated with OS and DFS rates.

**Table 1:** The relationship between BTG3 expression and clinicopathological features of colon carcinomas

Variables	Total number	BTG3 expression		
		MOD	SD	*p* value
Gender				0.599
Male	78	0.270	0.056	
Female	62	0.264	0.061	
Age (years)				0.649
< 62	70	0.269	0.055	
≥ 62	70	0.265	0.061	
Tumor location				0.991
Right sided	93	0.267	0.056	
Transverse sided	6	0.268	0.070	
Left sided	41	0.268	0.063	
Pathological classification				0.037*
Well/moderately differentiated adenocarcinoma	118	0.271	0.060	
Poorly differentiated/mucinous adenocarcinoma	22	0.247	0.044	
Depth of invasion				0.016*
T1-2	10	0.300	0.037	
T3-4	130	0.265	0.059	
Lymphatic invasion				0.101
Negative	82	0.274	0.055	
Positive	58	0.258	0.062	
Distant metastasis				0.024*
MO	135	0.269	0.058	
M1	5	0.210	0.030	
TNM staging				0.007*
I	6	0.318	0.014	
II	75	0.271	0.055	
III	55	0.261	0.061	
IV	4	0.203	0.029	
Vascular invasion				
No	118	0.269	0.059	0.448
Yes	22	0.259	0.053	
Nerve invasion				
No	109	0.268	0.056	0.718
Yes	31	0.264	0.066	

*SD* standard deviation, *MOD* mean optical density

*p < 0.05

**Table 2:** Univariate and multivariate analyses of individual parameters for correlation with OS or DFS rate

Variables	OS	*p* value	DFS	*p* value
	Relative risk (95% CI)		Relative risk (95% CI)	
Univariate analysis				
BTG3 MOD				
(> 0.2285 *vs* < 0.2285) for OS	0.338 (0.154–0.741)	0.007*		
(≥ 0.2345 *vs* < 0.2345) for DFS			0.305 (0.143–0.651)	0.002*
Differentiation (poorly vs well/moderate)	1.094 (0.375–3.187)	0.870	0.995 (0.345–2.870)	0.993
Pathological classification (mucinous vs tubular)	1.827 (0.625–5.337)	0.271	1.558 (0.540–4.493)	0.412
Depth of invasion (T3-4 vs T1-2)	1.748 (0.236–12.945)	0.584	0.969 (0.230–4.088)	0.965
Lymphatic invasion (positive vs negative)	2.667 (1.178–6.037)	0.019*	2.398 (1.123–5.121)	0.024*
Distant metastasis (positive vs negative)	1.043 (0.141–7.714)	0.967	0.962 (0.131–7.080)	0.970
TNM staging (III/IV vs I/II)	2.600 (1.149–5.884)	0.022*	2.321 (1.087–4.956)	0.030*
Vascular invasion (positive vs negative)	1.166 (0.400-3.401)	0.779	0.961 (0.333–2.771)	0.942
Nerve invasion (positive vs negative)	1.167 (0.466–2.923)	0.742	0.980 (0.397–2.418)	0.966
Multivariate analysis				
BTG3 MOD	0.378 (0.171–0.837)	0.016*		
(≥ 0.2285 vs < 0.2285) for OS				
(≥ 0.2345 vs < 0.2345) for DFS			0.331 (0.154–0.710)	0.005*
Differentiation (poorly vs well/moderate)	NA		NA	
Pathological classification (mucinous vs tubular)	NA		NA	
Depth of invasion (T3-4 vs T1-2)	NA		NA	
Lymphatic invasion (positive vs negative)	2.362 (1.035–5.39)	0.041*	2.131 (0.992–4.574)	0.047*
Distant metastasis (positive vs negative)	NA		NA	
TNM staging (III/IV vs I/II)	NA		NA	
Vascular invasion (positive vs negative)	NA		NA	
Nerve invasion (positive vs negative)	NA		NA	

*OS* overall survival, *DFS* disease-free survival, *95% CI* 95% confidence interval, *MOD* mean optical density, *NA* not applicable

*p < 0.05

### Loss of BTG3 promotes proliferation in the G2 phase, inhibits apoptosis, and promotes migration and invasion of CRC cell lines

**Fig. 2:**
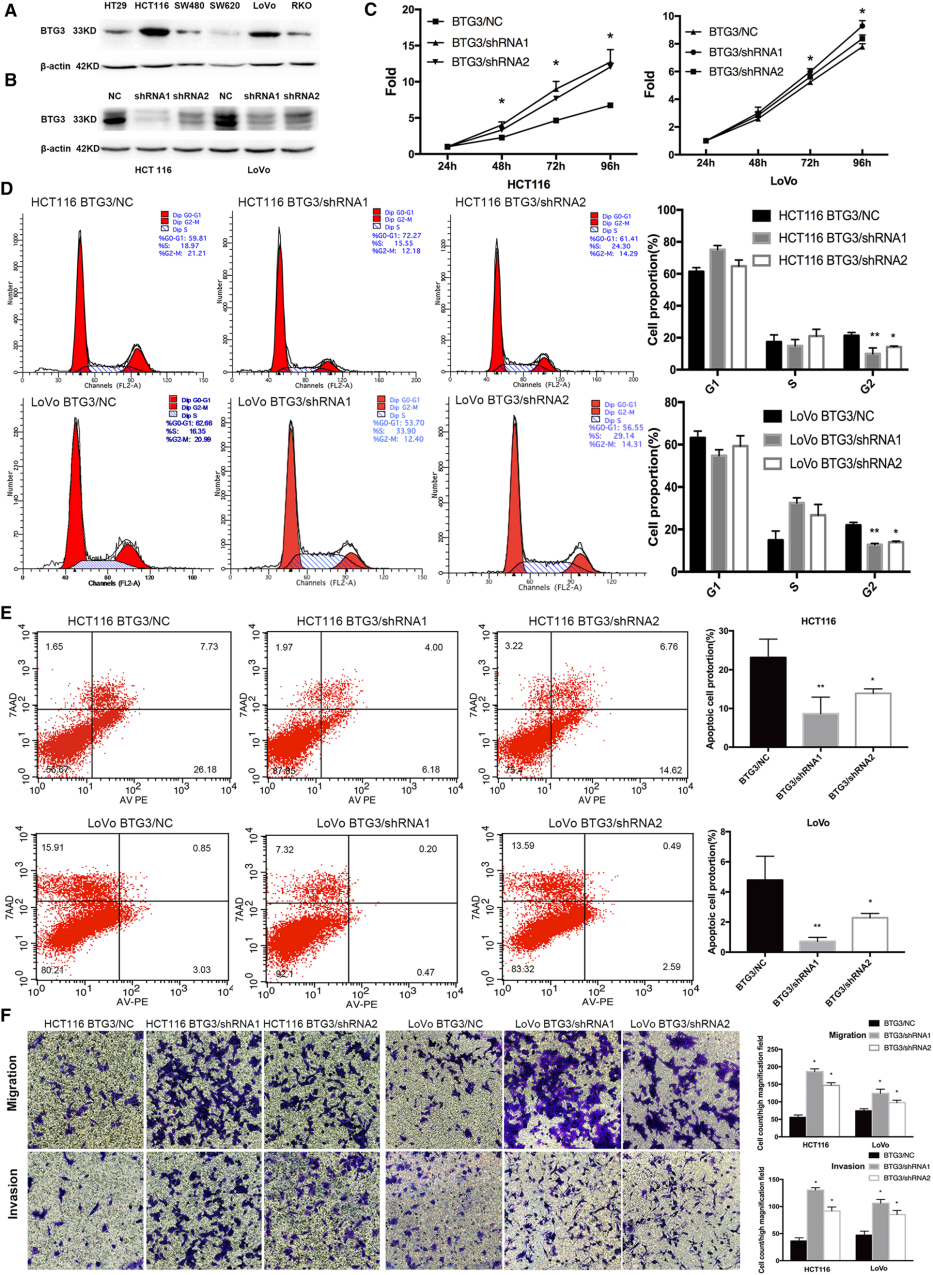
The effects of BTG3 knockdown on the phenotypes in CRC cells. **a** Western blotting analysis of BTG3 expression in six cell lines. **b** Western blotting analysis of BTG3 expression in HCT116 and LoVo cells with BTG3 knockdown. **c** Promotive effect of BTG3 knockdown on cell proliferation in vitro by the CCK-8 assay. **d** BTG3 knockdown decreased the percentage of G2 phase cells in the cell cycle transition. **e** Inhibitory effect of BTG3 knockdown on cell apoptosis. **f** HCT116 cells and LoVo cells with BTG3 knockdown showed an enhanced ability to migrate and invade. *p < 0.05, **p < 0.01, compared with that in the BTG3/NC group

To investigate the causative effects of BTG3 in the carcinogenesis of CRC, we first examined endogenous protein expression of BTG3 in HT29, HCT116, SW480, SW620, LoVo, and RKO cells by western blotting. HCT116 and LoVo cells exhibit relatively high expression of BTG3 (Fig. 2a) and were chosen for constructing the lentiviral-mediated small hairpin RNA (shRNA) cell lines. Cells were infected with lentivirus expressing BTG3/shRNA1 and shRNA2 or a silencing negative control sequence (NC/shRNA). The western blot results showed that BTG3/shRNA1 inhibited BTG3 expression with higher knockdown efficiency in the two cell lines (Fig. 2b). The CCK8 assay showed that the fold changes in OD450 values with BTG3/shRNA increased significantly in a time-dependent manner, compared with the control group. The promotive role of proliferation in the BTG3/shRNA1 group seemed more obvious than that in the BTG3/shRNA2 group (Fig. 2c). The deregulated cell cycle in tumors permits sustaining cell proliferation. Flow cytometry was conducted to further validate whether the promotive effect of BTG3 knockdown on CRC cell proliferation was mediated by a specific stage of the cell cycle. The results revealed that the BTG3/shRNA groups in both HCT116 and LoVo cells showed a significant decrease in the percentage of cells in the G2 peak compared with the NC group (BTG3/shRNA1 *p* < 0.01; BTG3/shRNA2 *p* < 0.05, Fig. 2d). These results suggest that BTG3 knockdown promoted CRC cell proliferation by relieving G2 phase arrest. Next, we determined the effect of BTG3 knockdown on apoptosis with Annexin V-PE/7AAD and flow cytometry. Cells in the lower right quadrant represented late apoptotic cells (Annexin V-PE positive/7-AAD positive). The rate of late apoptotic BTG3-depleted cells decreased significantly compared to that of the control, especially in the BTG3/shRNA1 group, suggesting that BTG3 knockdown inhibited CRC cell apoptosis (Fig. 2e). The effect of BTG3 knockdown on migration and invasion of HCT116 and LoVo cells was explored in Transwell assays. Significantly more HCT116 and LoVo cells migrated and invaded in the BTG3/shRNA group than that in the control group and the increase in the BTG3/shRNA1 group was more significant than that in BTG3/shRNA2 group (Fig. 2f). These results suggest that loss of BTG3 promoted migration and invasion of the CRC cell lines.

### Interruption of multiple critical pathways involved in cancer development by BTG3 knockdown

**Fig. 3:**
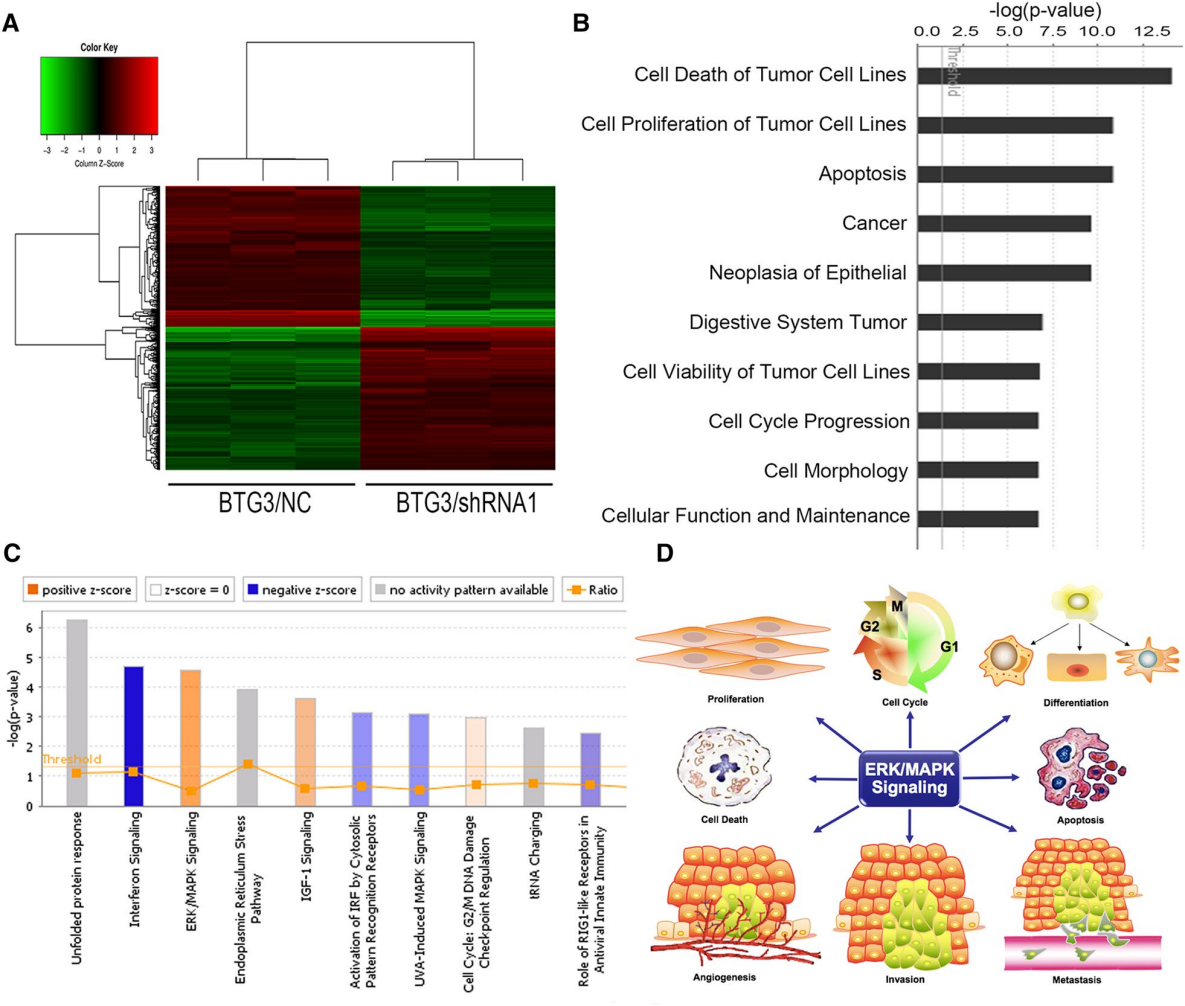
Microarray analysis and Ingenuity Pathway Analysis (IPA) of HCT 116 cells with BTG3 knockdown. **a** Heatmap representation of genes that were significantly differentially expressed in BTG3 cells infected with lentivirus expressing either BTG3/shRNA or NC/shRNA. Genes and samples are listed in rows and columns, respectively. A color scale for the normalized expression data is shown at the bottom of the microarray heatmap (red represents upregulated genes and green represents downregulated genes). **b** The IPA system revealed several dysregulated cellular and molecular functions. Here, the top ten diseases and functions were sorted in descending order by the inverse log of the *p* value. **c** Histogram shows the enrichment of differentially expressed genes in the classical signaling pathways. Activated (orange) and inhibited (blue) canonical pathways based on IPA databases were sorted in descending order by the inverse log of the *p* value. **d** The schematic diagram showed several major roles of the extracellular-regulated kinase/mitogen-activated kinase (ERK/MAPK) signaling pathway

**Fig. 4:**
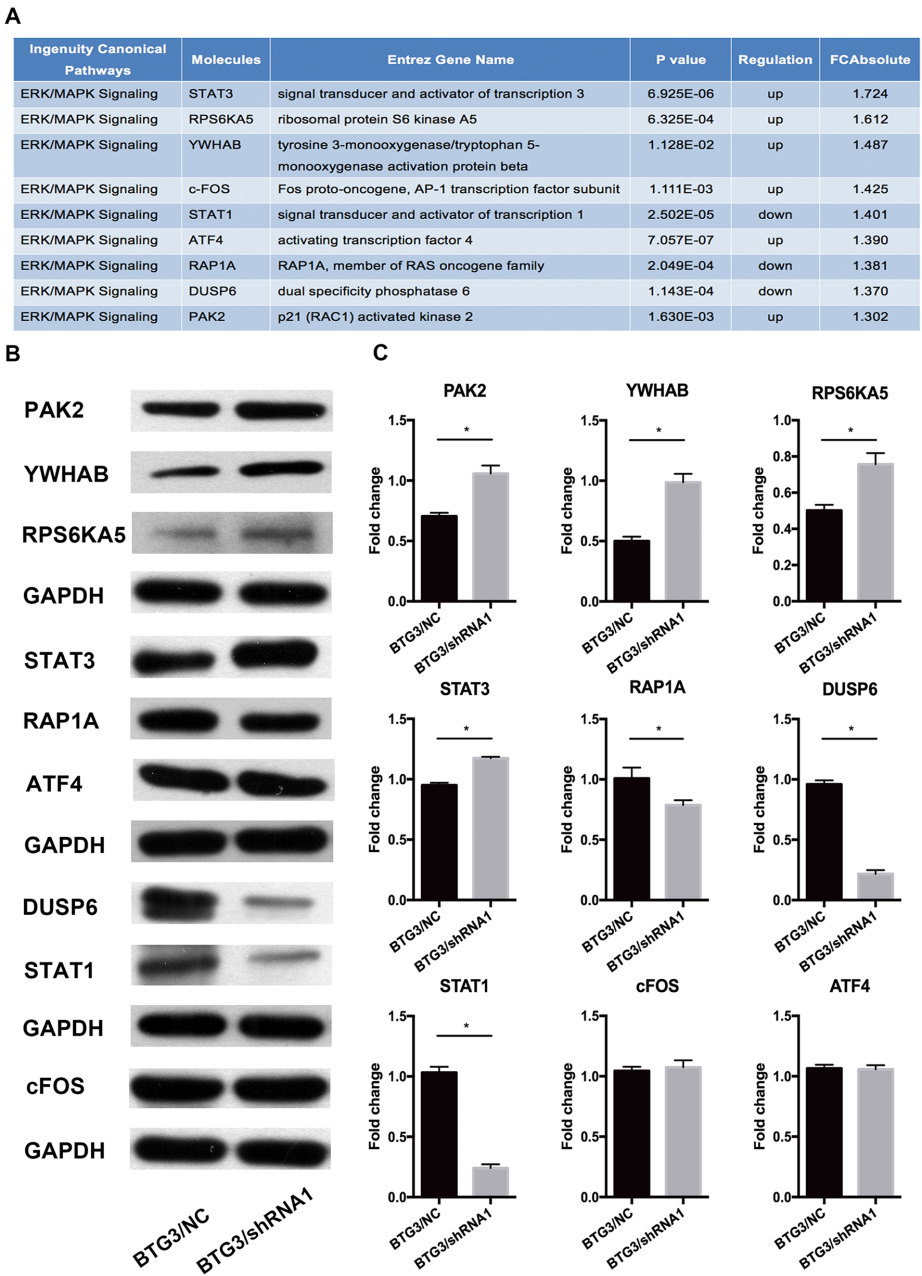
Western blotting validation of downstream genes indicated by the microarray and Ingenuity Pathway Analysis (IPA) data in HCT116 cell lines. **a** IPA shows several molecules involved in extracellular-regulated kinase/mitogen-activated kinase (ERK/MAPK) signaling changed in response to BTG3 knockdown. **b** The differential expression of molecules was screened by western blotting. **c** Representative histograms depicting validation of the involved proteins. *p < 0.05, compared with the BTG3/negative control (NC) group

The results revealed that decreased BTG3 expression is linked to carcinogenesis and aggressiveness of the CRC cell lines. However, the mechanisms underlying BTG3-mediated CRC development and its downstream pathways have not been systematically investigated. Here, genome-wide gene expression profiling of HCT116 cells infected with either BTG3/shRNA1 or NC/shRNA was analyzed using a microarray platform. In total, 554 genes were significantly differentially expressed (*p* < 0.05 and absolute fold change [FC absolute] > 1.3), among which 281 were upregulated and 273 were downregulated (Fig. 3a). Analysis of the microarray data using the Ingenuity Pathway Analysis (IPA) system showed several dysregulated cellular and molecular functions mediated by BTG3 knockdown, such as impaired cell death and survival, apoptosis, cellular growth and proliferation, cancer, cell cycle progression, and other disrupted cellular functions (Fig. 3b). The “canonical pathway” module was used to analyze the microarray data and to systematically explore the downstream pathways involved in cancer development, such as unfolded protein response signaling, endoplasmic reticulum stress signaling, and insulin-like growth factor-1 signaling (Fig. 3c). Among these impaired canonical pathways, extracellular-regulated kinase/mitogen-activated protein kinase (ERK/MAPK) signaling was highlighted. Furthermore, the IPA system listed several molecules involved in ERK/MAPK signaling mediated by BTG3 knockdown (Fig. 4a). Those differentially expressed genes were validated by western blot analysis in HCT116 cells and demonstrated that the PAK2, RPS6KA5, YWHAB, and signal transducer and activator of transcription (STAT)3 protein levels increased significantly, whereas those of RAP1A, DUSP6, and STAT1 clearly decreased in the BTG3/shRNA1 groups than those in the NC group. cFos and activating transcription factor 4 (ATF4) showed no conspicuous difference between the two groups (Fig. 4b). These results validated the gene expression profile results and are consistent with the microarray analysis.

## Discussion

Previous studies have indicated that BTG3 is a candidate tumor suppressor gene in various cancers (Lv et al. 2013; Lin et al. 2012; Majid et al. 2009; Gou et al. 2015). However, our knowledge of the expression patterns and function of BTG3 in the progression of CRC remains vague. In the present study, we first quantified BTG3 expression by comparing MOD values and found that BTG3 was significantly downregulated in CRC tissues compared with adjacent normal colorectal tissues, which indicated that downregulation of BTG3 may be associated with the progression of CRC. This result is consistent with other studies (Chen et al. 2013; Yu et al. 2008). Additionally, positively associations with BTG3 expression level and OS and DFS in patients with CRC and the multivariate analysis demonstrated that BTG3 expression was an independent factor indicating a favorable prognosis for CRC. A similar conclusion was reported in previous studies (Ren et al. 2015; Deng et al. 2013).

The positive correlation between BTG3 expression and clinicopathological characteristics suggested that BTG3 might be involved in CRC development and progression. To further clarify the roles of BTG3 in colorectal carcinogenesis and progression, we evaluated the effects of downregulating BTG3 on cell proliferation, the cell cycle, and apoptosis. Here, we observed that knockdown of BTG3 promoted CRC cell proliferation but inhibited CRC cell apoptosis. These results provide more support for an anticarcinogenic role in CRC and concur with those in hepatocellular carcinoma and gastric cancers (Lv et al. 2013; Ren et al. 2015). BTG3 dysregulates the S-phase by interacting with E2F1 to suppress binding of the E2F1-DP1 complex (Ou et al. 2007). As a key effector kinase in the cell cycle checkpoint response, checkpoint kinase 1 interacts with BTG3 to maintain genomic stability (Cheng et al. 2013). BTG3 overexpression induces S/G2 arrest in gastric cancer cells (Gou et al. 2015). Mao et al. (2016) reported that overexpression of BTG3 in CRC SW480 cells arrests the cell cycle at the G1 phase. In contrast, our studies show that downregulation of BTG3 in HCT16 and LoVo cells disturbed the cell cycle at the G2 phase. Different molecular phenotypes and epigenetic and genetic features may account for the different results. HCT116 cells are derived from Dukes’ D stage primary tumor, SW480 cells are derived from Dukes’ B stage primary tumor, and LoVo cells derived from Dukes’ C stage are from supraclavicular metastasis sites (Ahmed et al. 2013). Moreover, DNA methylation, point mutation, and microsatellite instability also differ in these cell lines (Ahmed et al. 2013).

The formation of regional invasion and distant metastasis is the main cause of morbidity and mortality in patients with cancer. Previous studies have shown that upregulation of BTG3 suppresses the migration and invasion capabilities of gastric cancer (Gou et al. 2015), and downregulation of BTG3 shows the opposite effect (Ren et al. 2015). As mentioned above in our study, patients with CRC and lower BTG3 expression levels had an unfavorable prognosis after the operation. Thus, we hypothesized that BTG3 might also play a role suppressing migration and invasion of CRC cells. Our results show that a deficiency of BTG3 remarkably promoted CRC cell migration and invasive abilities of CRC cells in vitro.

A critical role of BTG3 in CRC development and progression has been demonstrated in patient samples and in human CRC cell lines. However, molecular mechanism studies about the biological functions of BTG3 in CRC remain elusive. Here, a genomic microarray analysis was conducted in the HCT116 CRC cell line with BTG3 knocked down and hundreds of genes were significantly differentially expressed. The IPA further detected the gene signatures with disease functions and identified multiple pathways involved in cancer development and progression. For instance, the ERK/MAPK signaling pathway, which was the top-enriched pathway following BTG3 knockdown. This pathway possesses multiple functions in cancer cells, such as cell viability, proliferation and cell cycle progression (Morgan et al. 2001), apoptosis (Howie et al. 2008; Alejandro and Johnson 2008), differentiation (Dong et al. 2012), angiogenesis (Zeng et al. 2005; Huang et al. 2008), and migration and invasion (Weng et al. 2008; Lai et al. 2010) (Fig. 3d). It is also closely associated with the occurrence, development, metastasis, and prognosis of tumors and is an important reference value for clinical diagnosis and treatment (Balmanno et al. 2009; Zhang et al. 2009). Signal transduction along this pathway begins with activation of the small GTPases Ras or Rap by receptor tyrosine kinases and G-protein-coupled receptors (Kolch 2000). Next, the small G-proteins recruit c-Raf and B-Raf to the plasma membrane, where Raf is activated by binding to Ras (Kolch 2000). Then, MEK-1/2 becomes activated by Raf and further phosphorylates ERK-1/2 at threonine and tyrosine residues (Yung et al. 1997). Activated ERK regulates gene expression by directly phosphorylating transcription factors, such as Elk, and Myc, or indirectly by targeting substrates such as p90-RSK (ribosomal S6 kinase) family kinases (Davie and Spencer 2001).

The IPA revealed that the expression of nine genes (RAP1A, PAK2, YWHAB, RPS6KA5, STAT3, DUSP6, STAT1, cFosm, and ATF4) involved in ERK/MAPK signaling was altered after BTG3 knockdown. The protein expression levels of the nine differentially expressed mRNAs were verified by western blotting analysis, and were mainly in accordance with the microarray data. Ras-related protein Rap-1A is a protein encoded by the RAP1A gene. Rap1 primarily regulates multiple integrin-dependent processes, such as morphogenesis, cell–cell adhesion, hematopoiesis, leukocyte migration, and tumor invasion (Bos et al. 2001). Rap1 counteracts the mitogenic function of RAS because it competitively interacts with RAS GAPs and RAF (Han and Colicelli 1995). PAK2 is one of three members of the Group I PAK family of serine/threonine kinases. PAK2 signaling modulates apoptosis (Bokoch 1998) and cancers, including breast (Li et al. 2011), hepatocarcinoma (Sato et al. 2013), and gastric (Gao et al. 2014). Pak2 may phosphorylate c-Raf, ultimately resulting in activation of ERK (Hough et al. 2012). The YWHAB gene encodes the 14-3-3 protein, which is a beta/alpha protein in the 14-3-3 family of proteins. The 14-3-3 protein beta interacts with RAF1 and CDC25 phosphatases, suggesting that it may play a role linking mitogenic signaling and the cell cycle machinery (Qiu et al. 2000). The 14-3-3 protein beta regulates proliferation of hepatocellular carcinoma cells, tumor growth, and chemosensitivity via the MAPK pathway (Wu et al. 2015). MSK1 is a mitogen- and stress-activated protein kinase that is encoded by the RPS6KA5 gene and is activated by ERK and p38 MAPK in response to growth factors and cellular stress, respectively (Clark et al. 2007). MSK1 mediates histone H3 phosphorylation and immediate-early gene expression and transmits external signals into various responses involved in cancer development (Healy et al. 2012). Signal transducer and activator of transcription 3 (STAT3) is a transcription factor activated via phosphorylation of serine 727 by MAPK (Tkach et al. 2013). STAT3 plays a key role in many cellular processes, such as cell growth and apoptosis (Yuan et al. 2004). The protein dual specificity phosphatase 6 encoded by DUSP6 is a member of the dual specificity protein phosphatase subfamily (Muda et al. 1996). As a negative regulator of ERK, DUSP6 suppresses tumorigenesis and EMT-associated phenotypes (Wong et al. 2012). Contrary to STAT3 and STAT5, STAT1 is a tumor suppressor protein. Overexpression of STAT1 inhibits proliferation and migration but induces apoptosis in glioblastoma cells (Ju et al. 2013). ATF4 has also been isolated and characterized as cAMP response element binding protein 2 (CREB-2). It is involved in regulating transcription in a wide variety of cell types (Karpinski et al. 1992). Nerve growth factor, which activates a series of protein kinases via the ERK/MAPK signaling pathway, ultimately activates CREB (Sofroniew et al. 2001). The target genes mediated by CREB play an important role in cell proliferation, differentiation, survival, and the cell cycle. CREB overexpression promotes cell survival and proliferation and results in the development of various tumors (Sakamoto and Frank 2009). C-fos, referred to as an immediate early gene, is rapidly and transiently induced within 15 min of stimulation (Hu et al. 1994). Its activity is regulated by post-translational modification by various kinases, such as MAPK (Hurd et al. 2002). Our validation by western blotting indicated that PAK2, RPS6KA5, YWHAB, STAT3 were upregulated when BTG3 was depleted. In contrast, RAP1A, DUSP6, and STAT1 were downregulated in the BTG3/shRNA group compared with the NC group in HCT116 cells. This result might account for the phenotypes that downregulation of BTG3 expression could promote proliferation, inhibit apoptosis, and enhance invasion and metastasis of CRC cells. Interestingly, both cFos and ATF4 expressions did not change significantly. One possible reason may be that they are regulated by post-translational modification, such as phosphorylation. Nevertheless, our protein validation results implicate that the causal effect of BTG3 in CRC development and function may occur through regulation of the molecules mentioned above that are involved in ERK/MAPK signaling. The details of how BTG3 specifically interact with these molecules will be carried out in further studies.

In conclusion, our study confirmed the importance of BTG3 in CRC carcinogenesis and development and BTG3 might be considered a good biomarker for CRC prognosis. BTG3 knockdown in CRC cells promoted cell proliferation, migration, and invasion, disturbed the cell cycle, and impaired apoptosis by regulating multiple genes and cancer-associated pathways.

## References

[CR1] Ahmed D, Eide PW, Eilertsen IA, Danielsen SA, Eknaes M, Hektoen M (2013). Epigenetic and genetic features of 24 colon cancer cell lines. Oncogenesis.

[CR2] Alefan Q, Malhees R, Mhaidat N (2017). Direct medical cost associated with colorectal cancer in north of Jordan. Curr Probl Cancer.

[CR3] Alejandro EU, Johnson JD (2008). Inhibition of Raf-1 alters multiple downstream pathways to induce pancreatic beta-cell apoptosis. J Biol Chem.

[CR4] Balmanno K, Chell SD, Gillings AS, Hayat S, Cook SJ (2009). Intrinsic resistance to the MEK1/2 inhibitor AZD6244 (ARRY-142886) is associated with weak ERK1/2 signalling and/or strong PI3K signalling in colorectal cancer cell lines. Int J Cancer.

[CR5] Bokoch GM (1998). Caspase-mediated activation of PAK2 during apoptosis: proteolytic kinase activation as a general mechanism of apoptotic signal transduction?. Cell Death Differ.

[CR6] Bos JL, de Rooij J, Reedquist KA (2001). Rap1 signalling: adhering to new models. Nat Rev Mol Cell Biol.

[CR7] Budczies J, Klauschen F, Sinn BV, Gyorffy B, Schmitt WD, Darb-Esfahani S (2012). Cutoff finder: a comprehensive and straightforward web application enabling rapid biomarker cutoff optimization. PLoS One.

[CR8] Cassidy S, Syed BA (2017). Colorectal cancer drugs market. Nat Rev Drug Discov.

[CR9] Chen X, Chen G, Cao X, Zhou Y, Yang T, Wei S (2013). Downregulation of BTG3 in non-small cell lung cancer. Biochem Biophys Res Commun.

[CR10] Cheng YC, Lin TY, Shieh SY (2013). Candidate tumor suppressor BTG3 maintains genomic stability by promoting Lys63-linked ubiquitination and activation of the checkpoint kinase CHK1. Proc Natl Acad Sci USA.

[CR11] Clark CJ, McDade DM, O’Shaughnessy CT, Morris BJ (2007). Contrasting roles of neuronal Msk1 and Rsk2 in Bad phosphorylation and feedback regulation of Erk signalling. J Neurochem.

[CR12] Davie JR, Spencer VA (2001). Signal transduction pathways and the modification of chromatin structure. Prog Nucleic Acid Res Mol Biol.

[CR13] Deng B, Zhao Y, Gou W, Chen S, Mao X, Takano Y (2013). Decreased expression of BTG3 was linked to carcinogenesis, aggressiveness, and prognosis of ovarian carcinoma. Tumour Biol.

[CR14] Dong YC, Han QL, Zou Y, Deng ZL, Lu XL, Wang XH (2012). Long-term exposure to imatinib reduced cancer stem cell ability through induction of cell differentiation via activation of MAPK signaling in glioblastoma cells. Mol Cell Biochem.

[CR15] Du Y, Liu P, Zang W, Wang Y, Chen X, Li M (2015). BTG3 upregulation induces cell apoptosis and suppresses invasion in esophageal adenocarcinoma. Mol Cell Biochem.

[CR16] Gao C, Ma T, Pang L, Xie R (2014). Activation of P21-activated protein kinase 2 is an independent prognostic predictor for patients with gastric cancer. Diagn Pathol.

[CR17] Gou WF, Yang XF, Shen DF, Zhao S, Liu YP, Sun HZ (2015). The roles of BTG3 expression in gastric cancer: a potential marker for carcinogenesis and a target molecule for gene therapy. Oncotarget.

[CR18] Han L, Colicelli J (1995). A human protein selected for interference with Ras function interacts directly with Ras and competes with Raf1. Mol Cell Biol.

[CR19] Healy S, Khan P, He S, Davie JR (2012). Histone H3 phosphorylation, immediate-early gene expression, and the nucleosomal response: a historical perspective. Biochem Cell Biol.

[CR20] Hough C, Radu M, Dore JJ (2012). Tgf-beta induced Erk phosphorylation of smad linker region regulates smad signaling. PLoS One.

[CR21] Howie HL, Shiflett SL, So M (2008). Extracellular signal-regulated kinase activation by Neisseria gonorrhoeae downregulates epithelial cell proapoptotic proteins Bad and Bim. Infect Immun.

[CR22] Hu E, Mueller E, Oliviero S, Papaioannou VE, Johnson R, Spiegelman BM (1994). Targeted disruption of the c-fos gene demonstrates c-fos-dependent and -independent pathways for gene expression stimulated by growth factors or oncogenes. EMBO J.

[CR23] Huang D, Ding Y, Luo WM, Bender S, Qian CN, Kort E (2008). Inhibition of MAPK kinase signaling pathways suppressed renal cell carcinoma growth and angiogenesis in vivo. Can Res.

[CR24] Hurd TW, Culbert AA, Webster KJ, Tavare JM (2002). Dual role for mitogen-activated protein kinase (Erk) in insulin-dependent regulation of Fra-1 (fos-related antigen-1) transcription and phosphorylation. Biochem J.

[CR25] Ju H, Li X, Li H, Wang X, Wang H, Li Y (2013). Mediation of multiple pathways regulating cell proliferation, migration, and apoptosis in the human malignant glioma cell line U87MG via unphosphorylated STAT1: laboratory investigation. J Neurosurg.

[CR26] Karpinski BA, Morle GD, Huggenvik J, Uhler MD, Leiden JM (1992). Molecular cloning of human CREB-2: an ATF/CREB transcription factor that can negatively regulate transcription from the cAMP response element. Proc Natl Acad Sci USA.

[CR27] Kolch W (2000). Meaningful relationships: the regulation of the Ras/Raf/MEK/ERK pathway by protein interactions. Biochem J.

[CR28] Lai KC, Huang AC, Hsu SC, Kuo CL, Yang JS, Wu SH (2010). Benzyl isothiocyanate (BITC) inhibits migration and invasion of human colon cancer HT29 cells by inhibiting matrix metalloproteinase-2/-9 and urokinase plasminogen (uPA) through PKC and MAPK signaling pathway. J Agric Food Chem.

[CR29] Li X, Wen W, Liu K, Zhu F, Malakhova M, Peng C (2011). Phosphorylation of caspase-7 by p21-activated protein kinase (PAK) 2 inhibits chemotherapeutic drug-induced apoptosis of breast cancer cell lines. J Biol Chem.

[CR30] Lin TY, Cheng YC, Yang HC, Lin WC, Wang CC, Lai PL (2012). Loss of the candidate tumor suppressor BTG3 triggers acute cellular senescence via the ERK-JMJD3-p16(INK4a) signaling axis. Oncogene.

[CR31] Lv Z, Zou H, Peng K, Wang J, Ding Y, Li Y (2013). The suppressive role and aberrent promoter methylation of BTG3 in the progression of hepatocellular carcinoma. PLoS One.

[CR32] Majid S, Dar AA, Ahmad AE, Hirata H, Kawakami K, Shahryari V (2009). BTG3 tumor suppressor gene promoter demethylation, histone modification and cell cycle arrest by genistein in renal cancer. Carcinogenesis.

[CR33] Majid S, Dar AA, Shahryari V, Hirata H, Ahmad A, Saini S (2010). Genistein reverses hypermethylation and induces active histone modifications in tumor suppressor gene B-Cell translocation gene 3 in prostate cancer. Cancer.

[CR34] Mao D, Qiao L, Lu H, Feng Y (2016). B-cell translocation gene 3 overexpression inhibits proliferation and invasion of colorectal cancer SW480 cells via Wnt/beta-catenin signaling pathway. Neoplasma.

[CR35] Morgan MA, Dolp O, Reuter CW (2001). Cell-cycle-dependent activation of mitogen-activated protein kinase kinase (MEK-1/2) in myeloid leukemia cell lines and induction of growth inhibition and apoptosis by inhibitors of RAS signaling. Blood.

[CR36] Muda M, Boschert U, Dickinson R, Martinou JC, Martinou I, Camps M (1996). MKP-3, a novel cytosolic protein-tyrosine phosphatase that exemplifies a new class of mitogen-activated protein kinase phosphatase. J Biol Chem.

[CR37] Ou YH, Chung PH, Hsu FF, Sun TP, Chang WY, Shieh SY (2007). The candidate tumor suppressor BTG3 is a transcriptional target of p53 that inhibits E2F1. EMBO J.

[CR38] Qiu W, Zhuang S, von Lintig FC, Boss GR, Pilz RB (2000). Cell type-specific regulation of B-Raf kinase by cAMP and 14–3-3 proteins. J Biol Chem.

[CR39] Ren XL, Zhu XH, Li XM, Li YL, Wang JM, Wu PX (2015). Down-regulation of BTG3 promotes cell proliferation, migration and invasion and predicts survival in gastric cancer. J Cancer Res Clin Oncol.

[CR40] Sakamoto KM, Frank DA (2009). CREB in the pathophysiology of cancer: implications for targeting transcription factors for cancer therapy. Clin Cancer Res.

[CR41] Sato M, Matsuda Y, Wakai T, Kubota M, Osawa M, Fujimaki S (2013). P21-activated kinase-2 is a critical mediator of transforming growth factor-beta-induced hepatoma cell migration. J Gastroenterol Hepatol.

[CR42] Siegel R, Desantis C, Jemal A (2014). Colorectal cancer statistics, 2014. CA Cancer J Clin.

[CR43] Sofroniew MV, Howe CL, Mobley WC (2001). Nerve growth factor signaling, neuroprotection, and neural repair. Annu Rev Neurosci.

[CR44] Tkach M, Rosemblit C, Rivas MA, Proietti CJ, Diaz Flaque MC, Mercogliano MF (2013). p42/p44 MAPK-mediated Stat3Ser727 phosphorylation is required for progestin-induced full activation of Stat3 and breast cancer growth. Endocr Relat Cancer.

[CR45] Varghese C, Shin HR (2014). Strengthening cancer control in China. Lancet Oncol.

[CR46] Weng CJ, Chau CF, Hsieh YS, Yang SF, Yen GC (2008). Lucidenic acid inhibits PMA-induced invasion of human hepatoma cells through inactivating MAPK/ERK signal transduction pathway and reducing binding activities of NF-kappaB and AP-1. Carcinogenesis.

[CR47] Winkler GS (2010). The mammalian anti-proliferative BTG/Tob protein family. J Cell Physiol.

[CR48] Wolfgang CL (2013). Role of fourth-generation troponin in predicting mortality in noncardiac surgery. JAMA Surg.

[CR49] Wong VC, Chen H, Ko JM, Chan KW, Chan YP, Law S (2012). Tumor suppressor dual-specificity phosphatase 6 (DUSP6) impairs cell invasion and epithelial–mesenchymal transition (EMT)-associated phenotype. Int J Cancer.

[CR50] Wu YJ, Jan YJ, Ko BS, Liang SM, Liou JY (2015). Involvement of 14-3-3 proteins in regulating tumor progression of hepatocellular carcinoma. Cancers (Basel).

[CR51] Yu J, Zhang Y, Qi Z, Kurtycz D, Vacano G, Patterson D (2008). Methylation-mediated downregulation of the B-cell translocation gene 3 (BTG3) in breast cancer cells. Gene Expr.

[CR52] Yuan ZL, Guan YJ, Wang L, Wei W, Kane AB, Chin YE (2004). Central role of the threonine residue within the p+1 loop of receptor tyrosine kinase in STAT3 constitutive phosphorylation in metastatic cancer cells. Mol Cell Biol.

[CR53] Yung Y, Dolginov Y, Yao Z, Rubinfeld H, Michael D, Hanoch T (1997). Detection of ERK activation by a novel monoclonal antibody. FEBS Lett.

[CR54] Zeng QH, Li SL, Chepeha DB, Giordano TJ, Li J, Zhang HL (2005). Crosstalk between tumor and endothelial cells promotes tumor angiogenesis by MAPK activation of Notch signaling. Cancer Cell.

[CR55] Zhang HH, Walker F, Kiflemariam S, Whitehead RH, Williams D, Phillips WA (2009). Selective inhibition of proliferation in colorectal carcinoma cell lines expressing mutant APC or activated B-Raf. Int J Cancer.

